# Development of a Multiplex PCR Test with Automated Genotyping Targeting E7 for Detection of Six High-Risk Human Papillomaviruses

**DOI:** 10.1371/journal.pone.0130226

**Published:** 2015-06-18

**Authors:** Eliana Ferreira Paes, Angela Maria de Assis, Cirbia S. Campos Teixeira, Francisco Hideo Aoki, Julio Cesar Teixeira

**Affiliations:** 1 Department of Obstetrics and Gynecology, Faculty of Medical Sciences, University of Campinas (UNICAMP), Campinas, Sao Paulo, Brazil; 2 Laboratory of Research in AIDS, University of Campinas (UNICAMP), Campinas, Sao Paulo, Brazil; Queen Mary Hospital, HONG KONG

## Abstract

Cervical cancer is caused by high-risk human papillomaviruses (HPV) and viral detection tests aid in the diagnosis of precursor lesions. In the present study, a molecular test for detection of high-risk HPV DNA, called E7-HPV, was standardized and assessed in samples from women with pre-cancerous lesions. The development of the E7-HPV test for detection and genotyping of six high-risk HPV (types 16, 18, 31, 33, 45 and 52), consisted of evaluating primer quality and adjusting the multiplex PCR conditions. Primer design was based on the E7 region of each HPV, and the fluorochrome 6-FAM was added to PCR primers. Viral detection was performed by capillary electrophoresis in automated sequencer in samples obtained from 60 women (55 with ASC-H/HSIL cytology) from August to September 2013. A non-inferiority analysis was conducted with the cobas HPV test as a reference and following international guidelines for the development of new tests. The two tests had a high concordance rate in HPV16 detection (kappa=0.972), with only one discordant case (cervical intraepithelial neoplasia grade 3, negaive with cobas and positive for HPV16 by E7-HPV) and complete agreement in HPV18 detection. When comparing detection of all high-risk HPV, three cases were positive with cobas but negative with E7-HPV, and another three cases were negative with cobas but positive with E7-HPV (HPV16, 31 and 52). When we evaluate the cases initially suspected by cytology, the two tests had the same sensitivity in detection CIN2 or worse. In conclusion, the E7-HPV test has satisfactory initial results, and its development can be continued.

## Introduction

Cervical cancer is caused by persistent infection with high-risk human papillomaviruses (hrHPV) [1, 2]. The presence of hrHPV DNA in brush cervical samples can be detected by molecular tests and used to screen for women at risk. These DNA tests can help to identify high-grade cervical intraepithelial neoplasia (CIN) more efficiently than traditional cytology only, and some tests have been approved for screening [3–5].

Validation of new tests used for cervical cancer screening may be achieved by demonstrating noninferiority of clinical performance, in comparison to tests that were validated in large-scale high-cost studies, such as those performed for Hybrid Capture 2 (HC2) [6]. With this purpose, in 2009 experts in HPV-DNA tests and cervical cancer screening proposed international guidelines on the evaluation of a clinical equivalence of a new test [7].

The majority of existing HPV-DNA assays target region L1 of the virus, while some target regions E6 or E7 [8, 9]. In this study, we describe the development of a new test (E7-HPV) that uses multiplex PCR (m-PCR) with fluorochrome-labeled primers, based on region E7, for DNA detection and genotyping of six hrHPV (types 16, 18, 31, 33, 45 and 52).

## Materials and Methods

Samples obtained from 60 women seeking care from August to September 2013 in outpatient facilities at the Women’s Hospital (CAISM, Unicamp) were used. Of these women, 55 were ASC-H or HSIL on cytology and 5 had no cytological abnormalities. By histology, 49/55 had a diagnosis of CIN2 or worse (including 53/55 with CIN1 or worse) at the time of sample collection. The remaining 7 were considered normal.

Cervical samples for molecular testing were collected before colposcopy or loop electrosurgical excision procedure, and were obtained using routine nylon cytobrushes, applied in a rotating motion in the external cervical os. Subsequently, immersion and agitaton of the brush was performed in a tube with preservation medium for 10 seconds. Two samples were collected per patient: Surepath was used for the reference test (see below) and was maintained at 2–8°C, and phosphate buffered saline was used for the E7-HPV test, and was maintained at either 2–8°C or −20°C.

The reference test used for comparison with the E7-HPV test was the cobas HPV test (Roche Diagnostics, Pleasanton, CA, USA). The HC2 test that was advised by original guidelines for the evaluation of candidate tests, but we chose the cobas HPV test because the cobas test was evaluated in studies with thousands of women, demonstrated a higher accuracy than HC2, and was approved by the FDA in 2011. In 2014, the cobas HPV test was approved as a primary screening test for women 25 years or older [5]. The cobas test also perform HPV16 and 18 genotyping, wich allows for a direct comparison of the identification of HPV16 and 18 types by the cobas and E7-HPV tests.

### DNA extraction

Samples were centrifuged at 13.000 rpm for 10 min, the supernatant was removed, and 200 μl of digestion solution (1mM Tris pH 7.5 200 μg/ml of proteinase K, 0.5% SDS) was added to the cell pellet. The mixture was homogenized and incubated at 55°C for 2 h, followed by an incubation at 95°C for 5 min. Subsequently, 200 μl of phenol, chloroform, isoamyl solution (25:24:1) was added, the tube was shaken vigorously and centrifuged for 10 min at 12.000 rpm. The aqueous phase was removed and transferred to another tube, and 3 M sodium acetate (NaAc) with a pH of 5.2 was added in a proportion of 1:10. Absolute ethanol (2.5x the volume of the aqueous phase:NaAc) was added, the tube was agitated, and the solution was centrifuged at 13.000 rpm for 15 min. The supernatant was removed and 600 μl of 70% ethanol was added to the DNA pellet. Centrifugation was performed at 13.000 rpm for 10 min. The supernatant was removed and the pellet was resuspended in 20 μl of TE solution (1 mM Tris, 100 μM EDTA, pH 8.2) after all of the ethanol had evaporated. After 24 h of-refrigeration, the DNA was stored at -20°C until further testing.

### Quality control procedures

#### β-globin PCR

Adequate DNA extraction was confirmed by amplifying a 268 bp fragment of the human β-globin gene by conventional PCR, followed by agarose gel electrophoresis. All 60 samples tested positive for the β-globin gene.

#### DNA quantification

DNA concentration and purity were determined using a NanoDrop 1000 (Thermo Scientific) spectrophotometer. The optical density was read at 260–280 nm, and all 60 samples showed the expected concentration of 5–50 ng/μL and purity of 1.80.

The E7-HPV test was developed in the Laboratory of Biological Markers and Molecular Biology of Women’s Hospital, CAISM, Unicamp. All assays included negative controls that contained all reagents, except DNA, to monitor for potential contamination, and positive controls with DNA obtained from previously known samples. The DNA extracted from samples was tested by PCR by two electrophoresis techniques: 1.5% agarose gel (stained with 1 μg/ml ethidium bromide) and capillary electrophoresis. There was 100% concordance between the results of the two electrophoresis techniques.

### E7-HPV test

The test was developed for the detection and genotyping of six types of hrHPV: 16, 18, 31, 33, 45 and 52. For HPV16, the primer used was based on a publication by Walboomers et al. [1]. For the remaining five types, new primers were designed. The six pairs of primers were labeled with fluorochrome 6-FAM (carboxy fluorescein) and used in m-PCR, with product analysis performed by capillary electrophoresis, using an automated sequencer with genotype software. Details of the standardized method are described below.

### E7-HPV test primer design

During this study, with the exception of HPV16, new primers were designed with the GeneRunner program (version 3.05, Hastings Software). Constructionwas based on alignment of target sequences in the E7 gene region from each targeted human papillomavirus type; sequences were from the GenBank database (NCBI, NIH, USA). Primers ranged in length from 18 to 25 nucleotides. The size of the amplified products was predicted to vary from 100 bp to 400 bp. The interval for adequate melting temperature was defined, and no difference was observed in annealing temperature among the primers. Sense and anti-sense sequences were chosen so that the reaction would be free from possible dimers or intermolecular loop formation. All primers were tested singly with type-specific controls, and then in multiplex with type-specific controls. Cross-priming reactions were not observed. Primer sequences are shown in Table 1.

**Table 1:** Sequences of oligonucleotides labeled with fluorochrome 6-FAM (5’ primer sense) targeting the E7 region of HPV.

HPV type	Access number at Genbank^a^	Primer sequence (5’>3’) targeting E7 region	Position	Base pairs (n)
**16**	**NC_001526**	**5’ GATGAAATAGATGGTCCAGC 3’ (6FAM)**	**667–686**	**107**
	**NC_001526**	**5’ GCTTTGTACGCACAACCGAAGC 3’**	**752–774**	**107**
**18**	**NC_001357**	**5’ CGACGCAGAGAAACACAAGTAT 3’ (6FAM)**	**558–579**	**357**
	**NC_001357**	**5’ ATTGTTGCTTACTGCTGGGAT 3’**	**895–915**	**357**
**31**	**J04353.1**	**5’ GGCAACTGACCTCCACTG 3’ (6FAM)**	**613–630**	**228**
	**J04353.1**	**5’ ACAGTTGGGGCACACGATT 3’**	**823–841**	**228**
**33**	**PPH33CG**	**5’ CAGATGAGGATGAAGGCT 3’ (6FAM)**	**667–684**	**152**
	**PPH33CG**	**5’ GTAGTTGCTGTATGGTTCG 3’**	**801–819**	**152**
**45**	**EF202166**	**5’ AGGCACGGCAAGAAAGACT 3’ (6FAM)**	**532–550**	**309**
	**EF202166**	**5’ TCTAAGGTCATCTGCCGAGC 3’**	**822–841**	**309**
**52**	**GQ472848**	**5’ GACCTGTGACCCAAGTGTAAC 3’ (6FAM)**	**529–549**	**400**
	**GQ472848**	**5’ GCCTCTACTTCAAACCAGCC 3’**	**909–928**	**400**

^a^GenBank (http://www.ncbi.nlm.nih.gov/genbank).

### E7-HPV test PCR

The presence of DNA of HPV types 16, 18, 31, 33, 45 and 52 was detected by m-PCR amplification using type specific primers labeled with fluorochrome 6-FAM at the 5’-end. For m-PCR, 15 μl reagent mixtures contained 5 Units of Taq Platinum DNA polymerase, 0.25 mM deoxyribonucleotide (dNTPs), 10x concentrated buffer, 4 mM MgCl_2_, 0.2 pmol of each primer and 50 ng of DNA. In the initial denaturation phase, the temperature was 95°C for 2 min. Thirty-five amplification cycles were performed. Each cycle included denaturation at 95°C for 15 s, primer binding to DNA at 64°C for 20 s, and amplicon extension at 72°C for 1 min. In the final phase, the temperature was maintained at 72°C for 7 min for polymerization of incomplete fragments. Each primer was carefully designed to have the same annealing temperature (64°C).

### PCR product analysis by capillary electrophoresis

PCR product (1 μl) was added to a mixture of 9 μl formamide and 0.2 μl of molecular weight marker LIZ 500, before denaturation (85°C for 3 min). The specimen was introduced in the 24-capillary array ABI 3500XL (Applied Biosystems), with previous capillary loading with Polymer POP7 (minimum execution time: 18 min). The molecular weight marker compatible with this set is the GeneScan 500 LIZ (Invitrogen-Life Technologies) that has fragments ranging from 35 to 500 bp. Fragments with at least 70 bp are indicated to ensure a more accurate analysis. m-PCR with six HPV types was mixed with GeneScan 500 LIZ marker (Invitrogen-Life Technologies) in each plate orifice and run in the same capillary. This was possible because primers were labeled with fluorochrome 6-FAM, generating amplicons of different sizes for each HPV type (107, 357, 228, 152, 309 and 400 bp, Table 1). Sample injection was performed during 30 s with an electric current of 3 KV. The electrophoretic run was conducted for 20 min with an electric current of 9 KV. Data obtained from the 60 samples were analyzed by electropherogram interpretation, using GeneMapper software ID-X Version 1.2 (Life Technologies). Using the data output file, genetic analysis was carried out through visual comparison with standard sizes of amplicons. In the reaction products undergoing capillary electrophoresis, we visualized fluorescent peaks of different sizes, according to the amplicon size of each specific HPV type. Positive controls consisted of samples collected from patients previously and known to be infected with one HPV type. Positive control samples were processed under similar conditions. Negative controls consisted of water in place of the DNA PCR product (with all of the other PCR amplification reagents also present).

### Reference test: cobas HPV Test

This test is capable of detecting a combination of 12 hrHPV types (types 31, 33, 35, 39, 45, 51, 52, 56, 58, 59, 66 and 68), conducting individual genotyping for types 16 and 18, and identifying β-globin. The test extracts HPV DNA from cell samples and performs real-time PCR amplification, using specific primer pairs for β-globin and HPV. Amplicon detection is performed using probes stained with different fluorescent dyes. The 12 hrHPV are detected using the same fluorescent dye, while HPV16, HPV18, and β-globin are detected by different fluorophores. For this study, the cobas HPV test was performed in an outside laboratory, suggested by Roche Molecular Diagnostics, Brazil, using the cobas 4800 System.

### Statistical analysis

Following an international reference guideline [7], initial validation is achieved in a non-inferiority study when a new test demonstrates 90% of the sensitivity of the reference test for detecting CIN2 or worse. Presuming that equal sensitivity occurred between tests and there was a good agreement among results (kappa = 0.7), the statistical power is 80% to 90% when 50 to 60 samples are evaluated [7].

### Management of laboratory results and ethics statement

The E7-HPV test and the cobas HPV test were performed in two different laboratories. Researchers were blinded to laboratory results, clinical information, diagnosis, and patient identification. Results were compared only at the end of the studies and any discrepancies found were reviewed and analyzed either by repeat cobas HPV test to confirm negative discordant tests, complementary tests with known available primers, or genotyping of 35 HPV types by the CLART HPV2 test (Genomica) to identify viral discordant types among tests. These repeat and complementary tests were performed in the previously cited outside laboratory that was kept blinded to clinical information or previous test result. The study followed current regulatory standards of the National Council of Health of Brazil, and was approved by both the Institutional Review Board of the Women’s Hospital and for the Ethics Committee of the University of Campinas. The patients were informed about the study and signed a consent form to participate.

## Results

The E7-HPV test and cobas HPV test showed a high concordance of results for HPV16 detection with a kappa = 0.97 and only one discordant case. This case (case 28) had a CIN3 histopathologic lesion, a positive E7-HPV test, and a negative cobas HPV test (Tables 2 and 5). For HPV18, test results were totally concordant (kappa = 1.00, Table 2).

**Table 2:** Evaluation of concordance between E7-HPV test and cobas HPV test for the detection of HPV16 or 18, in 60 uterine cervical samples.

E7-HPV test	cobas HPV test		kappa test
	HPV16 (-)	HPV16 (+)	
**HPV16 (-)**	39	0	
**HPV16 (+)**	1	20	**0.97**
	**HPV18 (-)**	**HPV18 (+)**	
**HPV18 (-)**	54	0	
**HPV18 (+)**	0	6	**1.00**

For the detection of hrHPV other than HPV16/18, good concordance between E7-HPV and cobas was observed, with a kappa = 0.83. Six cases were positive for cobas and negative for the E7-HPV test. This may occur because cobas detects HPV types not included in the E7-HPV. In contrast, the E7-HPV test detected hrHPV in two cases in which the cobas HPV test was negative: cases 21 and 29 (Tables 3 and 5).

**Table 3:** Evaluation of concordance between E7-HPV test and cobas HPV test for hrHPV detection other than HPV type 16 and 18, in 60 uterine cervical samples.

E7-HPV test (HPV31, 33, 45, 52)	cobas HPV test (12 hrHPV^a^)		Total (n)
	HPV (-)	HPV (+)	
**HPV (-)**	32	6	38
**HPV (+)**	2	20	22
**Total**	34	26	60

kappa test = 0.83

hrHPV: high-risk Human Papillomavirus.

^a^ HPV31, 33, 35, 39, 45, 51, 52, 56, 58, 59, 66 and 68.

Overall sensitivity of both tests for detection of CIN2 or worse was 80% (Table 4), but case-to-case evaluation showed nine discordant situations, described in Table 5.

**Table 4:** Performance of the E7-HPV test and cobas HPV test in diagnosis of CIN.

Test		Final diagnosis (n = 60)			
		CIN1+		CIN2+	
		No (n = 7)	Yes (n = 53)	No (n = 11)	Yes (n = 49)
**cobas HPV test** (14 viral types)	**Negative**	*5*	*13*	*8*	*10^a^*
	**Positive**	*2*	*40*	*3^b^*	*39*
**E7-HPV test** (6 viral types)	**Negative**	*5*	*13*	*8*	*10^a^*
	**Positive**	*2*	*40*	*3^b^*	*39*

CIN1+ / CIN2+: cervical intraepithelial neoplasia grade 1 or worse / grade 2 or worse.

Sensitivity = 0.76 for both tests to detect CIN1+.

Sensitivity = 0.80 for both tests to detect CIN2+.

^a^ Three non-coincident cases among tests (Table 5).

^b^ Same cases for both tests.

**Table 5:** Discordant results between the E7-HPV test and cobas HPV test.

Case	Age (years)	Final Diagnosis	Cobas HPV test	E7-HPV test	Confirmatory test^a^
**21**	26	CIN2	(-)	HPV52 (+)	cobas(-)
**28**	43	CIN3	(-)	HPV16 (+)	cobas (-)
**29**	47	CIN3	(-)	HPV31 (+)	cobas (-)
**30**	40	CIN3	hrHPV (+) (non-HPV16/18)	(-)	CLART (-)
**34**	34	CIN2	hrHPV (+) (non-HPV16/18)	(-)	CLART HPV51 (+)
**52**	48	CIN2	hrHPV (+) (non-HPV16/18)	(-)	CLART HPV39 (+)
**31**	29	CIN3	HPV16 (+) and hrHPV (+)	HPV16 (+)	CLART HPV16 and 58 (+)
**37**	41	CIN3	HPV16 (+) and hrHPV (+)	HPV16 (+)	CLART HPV16 and 35 (+)
**58**	29	CIN3	HPV16 (+) and hrHPV (+)	HPV16 (+)	CLART HPV16 and 51 (+)

CIN: cervical intraepithelial neoplasia.

cobas HPV test (Roche): PCR Real Time with a sensitivity of 80 viral copies/ml for detection of 12 hrHPV combined (types 31, 33, 35, 39, 45, 51, 52, 56, 58, 59, 66 and 68) and individual genotyping of HPV 16 and 18.

CLART HPV2 test (Genomica): m-PCR with a sensitivity of 50–100 copies/samples for detection of 35 types of HPV (high-risk HPV-16, 18, 26, 31, 33, 35, 39, 45, 51, 52, 53, 56, 58, 59, 66, 68, 70, 73, 85 and low-risk HPV-6, 11, 40, 42, 43, 44, 54, 61, 62, 71, 72, 81, 82, 83, 84, 89).

^a^ tests performed in different aliquots of the same sample.

The E7-HPV test had a similar ability to detect compared with the cobas HPV test. In addition, the E7-HPV test detected hrHPV in three cobas negative cases: HPV52 (case 21), HPV16 (case 28) and HPV31 (case 29) (Table 5).

## Discussion

The E7-HPV test performs similarly to the cobas HPV test in detecting HPV16, HPV18, and other hrHPV types in samples from women with CIN2 or worse lesions. This test performance fulfils the initial prerequisites of the validation process.

The development of the E7-HPV test originated from the assumption that it was possible to produce a test that was easy to perform and that generated results relatively quickly at a low cost.

The majority of available tests detect a pool of hrHPV types in a single reaction, while some tests individually perform HPV16 and 18 genotyping [4, 6, 8]. The hrHPV types targeted by the E7-HPV test (types 16, 18, 31, 33, 45 and 52) were chosen because they are the most commonly associated with cervical cancer [10–14]. In particular, HPV52 has a high prevalence in the Brazilian population [11, 12]. The E7-HPV test characteristics result in adequate applicability in primary screening, while traditional cytology is expected to remain as second-line screening.

The E7 sequence was chosen for its relevance in oncogenesis and because it is relatively short and conserved sequence. This allows for the detection of viral DNA that is integrated into the host genome, which avoids false negative tests due to ruptures in region E1–L1 during the integrating process. L1 is the target of many of the commercially-available tests, including the cobas HPV test [1, 9].

Detailed type-specific primer design, except for HPV16, was performed in our laboratory. Fluorochrome 6-FAM was added to all primers, permitting automated reading in the DNA sequencer, eliminating any subjectivity in the evaluation of the results. This system was used because of the high discriminatory power among diverse HPV types that were combined and run in a single reaction [15, 16].

The E7-HPV test was carried out with automated capillary electrophoresis with 20 samples processed every 20 minutes. The amplification products were run in both capillary and agarose gel electrophoresis, and the results were 100% concordant. The E7-HPV and cobas tests were conducted in different laboratories in a blinded fashion to clinical information and test results. A case-to-case comparison was only made after all results were available.

The E7-HPV test performed adequately, and detected every HPV16 and 18 that the cobas HPV test detected (kappa≥0.97 and the same sensitivity for CIN2 or worse). In addition, the new test identified hrHPV in three CIN2 or worse cases (HPV16, 31 and 52) where the cobas HPV test was negative, including a confirmatory repeated test.

The main oncogenic HPV types are 16 and 18, prevalent in 50% of CIN2 or worse lesions and 70% of cervical cancer [4]. The remaining 12 hrHPV types are less prevalent in the etiology of cervical cancer, and are detected as a group by the cobas HPV test. In contrast, the E7-HPV test detects and individually genotypes four hrHPV types other than HPV16 and 18.

Identification of non-HPV16/18 hrHPV types also showed good concordance between the two tests (kappa = 0.83). There were three hrHPV positive non-16/18 cases identified by the cobas HPV test which were negative by the E7-HPV test. Two of these three occurred due to a higher number of hrHPV detected by the cobas HPV test (12 vs. 4), which was confirmed by genotyping by the CLART HPV2 test. The third case was negative by both CLART HPV2 and E7-HPV tests. This case could be explained by the differences in sensitivity between each test or samples analyzed, or differences in viral target region between tests.

One limitation of the E7-HPV test could be the smaller number of hrHPV types detectable. However, this is somewhat compensated by the genotyping offered for the six most important hrHPV types (16, 18, 31, 33, 45 and 52), according to the prevalence of each type in the total number of cervical cancers evaluated in samples worldwide [14]. It was necessary to define a composition for hrHPV detection that was useful and feasible, and other HPV types can be added for future genotyping.

Another advantage in genotyping is that it can be used in clinical practice to define persistent infection by the same HPV type in follow-up evaluation of women. Persistent infection is considered the major risk factor for the development and progression of precursor lesions to cervical cancer [2] and it cannot be definitely determined through tests that do not perform genotyping.

High performance and predicted low cost are strengths of the E7-HPV test. Access to many currently available tests is restricted, due to their high cost or lack of routine incorporation into official screening programs.

## Conclusion

This study defined a standard methodology for a new molecular test with capacity to identify and genotype six hrHPV genotypes in cervical samples from women with cervical pre-cancer lesions. The results fulfilled the prerequisites defined by international guidelines that recommend the development and analysis of new tests, with a high concordance and adequate sensitivity compared to the cobas reference test. The results indicate a potential future applicability of the new test and support the continued development of the E7-HPV test.

## Supporting Information

S1 Dataset Information about the cases and tests results. (PDF)
