# Supplementary material for: Development of a Multiplex PCR Test with Automated Genotyping Targeting E7 for Detection of Six High-Risk Human Papillomaviruses
Source: PLoS One. 2015 Jun 18;10(6):e0130226. doi: 10.1371/journal.pone.0130226 (PMC4472756; doi:10.1371/journal.pone.0130226)
Supplement: S1 Dataset — (PDF) [file pone.0130226.s001.pdf]

Case	Sample collection	Cytology		Colposcopy	Final histology		Final procedure	
	DD/MM/YYYY	DD/MM/YYYY	Result	DD/MM/YYYY	DD/MM/YYYY	Result	DD/MM/YYYY	Result
1	22/07/2013	01/03/2013	HSIL/ ASC-H	12/07/2013	22/07/2013	CIN 3	22/07/2013	LEEP
2	22/07/2013	10/04/2013	HSIL/ ASC-H	10/04/2013	22/07/2013	CIN 1	22/07/2013	LEEP
3	22/07/2013	15/03/2013	HSIL/ ASC-H	22/07/2013	22/07/2013	CIN 2	22/07/2013	LEEP
4	24/07/2013	06/03/2012	HSIL/ ASC-H	24/07/2013	13/05/2013	CIN 3	12/08/2013	LEEP
5	29/07/2013	01/03/2013	HSIL/ ASC-H	29/07/2013	29/07/2013	CIN 3	29/07/2013	LEEP
6	24/07/2013	16/07/2012	HSIL/ ASC-H	24/02/2013	29/07/2013	CIN 2	29/07/2013	LEEP
7	07/08/2013	07/08/2013	Negative	07/08/2013	Not done	Negative	NA	NA
8	31/07/2013	31/07/2013	HSIL/ ASC-H	31/07/2013	21/10/2013	CIN 3	21/10/2013	LEEP
9	05/08/2013	15/03/2013	HSIL/ ASC-H	17/06/2013	05/08/2013	CIN 3	05/08/2013	LEEP
10	14/08/2013	14/08/2013	Negative	14/08/2013	Not done	Negative	NA	NA
11	07/08/2013	29/05/2013	HSIL/ ASC-H	07/08/2013	18/11/2013	CIN 3	18/11/2013	LEEP
12	09/08/2013	13/07/2013	HSIL/ ASC-H	09/08/2013	Not done	Negative	NA	NA
13	16/08/2013	16/08/2013	Negative	16/08/2013	Not done	Negative	NA	NA
14	12/08/2013	05/07/2013	HSIL/ ASC-H	30/07/2013	12/08/2013	CIN 1	12/08/2013	LEEP
15	12/08/2013	01/04/2013	HSIL/ ASC-H	29/04/2013	12/08/2013	CIN 3	12/08/2013	LEEP
16	19/08/2013	18/02/2013	Negative	19/08/2013	Not done	Negative	NA	NA
17	12/08/2013	01/04/2013	HSIL/ ASC-H	09/08/2013	12/08/2013	Negative	12/08/2013	LEEP
18	12/08/2013	01/03/2013	HSIL/ ASC-H	14/06/2013	12/08/2013	CIN 3	12/08/2013	LEEP
19	16/08/2013	01/05/2013	Negative	16/08/2013	Not done	Negative	NA	NA
20	14/08/2013	15/06/2013	HSIL/ ASC-H	14/08/2013	19/11/2013	Microinvasor	10/01/2014	hysterectomy
21	19/08/2013	09/04/2013	HSIL/ ASC-H	09/04/2013	30/09/2013	CIN 2	19/08/2013	LEEP
22	19/08/2013	28/06/2013	HSIL/ ASC-H	28/06/2013	19/08/2013	CIN 2	19/08/2013	LEEP
23	19/08/2013	01/05/2013	HSIL/ ASC-H	26/08/2013	19/08/2013	CIN 1	19/08/2013	LEEP
24	19/08/2013	01/03/2013	HSIL/ ASC-H	01/05/2013	19/08/2013	CIN 3	19/082013	LEEP
25	19/08/2013	31/07/2013	HSIL/ ASC-H	31/07/2013	19/08/2013	CIN 3	13/09/2013	hysterectomy
26	21/08/2013	19/06/2013	HSIL/ ASC-H	21/08/2013	09/12/2013	CIN 3	09/12/2013	LEEP (CIN3)
27	21/08/2013	12/05/2013	HSIL/ ASC-H	21/08/2013	13/01/2014	CIN 2	13/01/2014	LEEP
28	23/08/2013	23/08/2013	HSIL/ ASC-H	23/08/2013	16/09/2013	CIN 3	16/09/2013	LEEP
29	26/08/2013	01/03/2013	HSIL/ ASC-H	06/08/2013	26/082013	CIN 3	26/082013	LEEP

Case	Sample collection	Cytology		Colposcopy	Final histology		Final procedure	
	DD/MM/YYYY	DD/MM/YYYY	Result	DD/MM/YYYY	DD/MM/YYYY	Result	DD/MM/YYYY	Result
30	26/08/2013	21/05/2013	HSIL/ ASC-H	14/05/2013	26/08/2013	CIN 3	26/08/2013	LEEP (CIN3)
31	26/08/2013	24/07/2013	HSIL/ ASC-H	06/08/2013	26/08/2013	CIN 3	26/08/2013	LEEP
32	28/08/2013	01/06/2013	HSIL/ ASC-H	28/08/2013	16/12/2013	CIN 3	16/12/2013	LEEP (CIN3)
33	28/08/2013	10/06/2013	HSIL/ ASC-H	28/08/2013	28/08/2013	CIN 2	28/08/2013	biopsia
34	30/08/2013	01/05/2013	HSIL/ ASC-H	30/08/2013	06/01/2014	CIN 2	06/01/2014	LEEP (CIN2)
35	02/09/2013	01/11/2012	HSIL/ ASC-H	22/04/2013	22/04/2013	CIN 3	22/04/2013	CIN 3
36	02/09/2013	15/05/2013	HSIL/ ASC-H	15/05/2013	02/09/2013	Microinvasor	10/01/2014	hysterectomy
37	02/09/2013	01/05/2013	HSIL/ ASC-H	02/07/2013	02/09/2013	CIN 3	02/09/2013	LEEP (CIN3)
38	04/09/2013	04/09/2013	HSIL/ ASC-H	04/09/2013	04/09/2013	CIN 2	04/09/2013	Biopsia
39	04/09/2013	01/02/2013	HSIL/ ASC-H	04/09/2013	04/11/2013	CIN 1	04/11/2013	LEEP
40	04/09/2013	01/11/2012	HSIL/ ASC-H	04/09/2013	22/11/2013	Invasor	22/11/2013	Radical hysterectomy
41	09/09/2013	14/05/2013	HSIL/ ASC-H	14/05/2013	09/09/2013	CIN 3	09/09/2013	LEEP
42	09/09/2013	09/05/2013	HSIL/ ASC-H	02/07/2013	09/09/2013	CIN 3	09/09/2013	LEEP
43	09/09/2013	23/05/2013	HSIL/ ASC-H	23/05/2013	09/09/2013	Microinvasor	16/01/2014	hysterectomy
44	09/09/2013	22/05/2013	HSIL/ ASC-H	01/09/2010	09/09/2013	CIN 3	09/09/2013	LEEP (CIN1)
45	09/09/2013	01/11/2012	HSIL/ ASC-H	28/08/2013	09/09/2013	CIN 3	09/09/2013	LEEP
46	11/09/2013	25/06/2013	Invasor	11/09/2013	05/12/2013	Invasor	05/12/2013	Cone frio
47	11/09/2013	12/06/2013	HSIL/ ASC-H	11/09/2013	04/11/2013	CIN 3	04/11/2013	LEEP
48	09/09/2013	17/05/2013	HSIL/ ASC-H	09/09/2013	25/11/2013	CIN 3	25/11/2013	LEEP
49	16/09/2013	01/04/2013	HSIL/ ASC-H	16/09/2013	16/09/2013	CIN 3	16/09/2013	LEEP
50	16/09/2013	13/04/2013	HSIL/ ASC-H	20/08/2013	16/09/2013	CIN 3	16/09/2013	LEEP
51	16/09/2013	08/05/2013	HSIL/ ASC-H	19/08/2013	16/09/2013	Microinvasor	16/09/2013	LEEP
52	16/09/2013	25/04/2013	HSIL/ ASC-H	02/09/2013	16/09/2013	CIN 2	16/09/2013	LEEP (CIN2)
53	18/09/2013	01/03/2013	HSIL/ ASC-H	18/09/2013	01/03/2013	CIN 2	01/03/2013	LEEP
54	23/09/2013	18/04/2013	HSIL/ ASC-H	23/09/2013	23/09/2013	CIN 3	23/09/2013	LEEP
55	23/09/2013	03/07/2013	HSIL/ ASC-H	03/07/2013	23/09/2013	CIN 3	23/09/2013	LEEP
56	23/09/2013	20/08/2012	HSIL/ ASC-H	23/09/2013	23/09/2013	CIN 3	23/09/2013	LEEP
57	23/09/2013	17/04/2013	HSIL/ ASC-H	23/09/2013	23/09/2013	CIN 3	23/09/2013	LEEP
58	23/09/2013	01/11/2012	HSIL/ ASC-H	23/09/2013	23/09/2013	CIN 3	23/09/2013	LEEP
59	30/09/2013	21/08/2012	HSIL/ ASC-H	01/01/2012	13/09/2013	CIN 3	30/09/2013	LEEP
60	30/09/2013	01/06/2013	HSIL/ ASC-H	03/09/2013	30/09/2013	CIN 3	30/09/2013	LEEP

	Case	Reference test (COBAS)			Study test (E7-HPV)			Confirmatory or Re-test
		12 hr-HPV	hpv 16	hpv 18	Agarose	type	Capillary	
	1	negative	negative	negative	negative		negative	
	2	negative	negative	negative	negative		negative	
	3	negative	negative	negative	negative		negative	
	4	positive	negative	negative	HR+ other than 16/18	31	HR+ other than 16/18	
	5	negative	positive	positive	16 e 18		16 e 18	
	6	positive	negative	negative	HR+ other than 16/18	52	HR+ other than 16/18	
	7	negative	negative	negative	negative		negative	
	8	positive	negative	negative	HR+ other than 16/18	33	HR+ other than 16/18	
	9	negative	negative	negative	negative		negative	
	10	negative	negative	negative	negative		negative	
	11	positive	negative	negative	HR+ other than 16/18	33	HR+ other than 16/18	
	12	negative	negative	negative	negative		negative	
	13	negative	negative	negative	negative		negative	
	14	negative	negative	positive	18		18	
	15	negative	negative	positive	18		18	
	16	negative	negative	negative	negative		negative	
	17	positive	negative	negative	HR+ other than 16/18	31	HR+ other than 16/18	
	18	positive	negative	negative	HR+ other than 16/18	33	HR+ other than 16/18	
	19	positive	negative	negative	HR+ other than 16/18	52	HR+ other than 16/18	
	20	negative	negative	positive	18		18	
	21	negative	negative	negative	HR+ other than 16/18	52	HR+ other than 16/18	Cobas neg/neg/neg
	22	positive	positive	negative	16 (33, 52)	33 52	16 (33, 52)	
	23	negative	negative	negative	negative		negative	
	24	positive	negative	negative	HR+ other than 16/18	52	HR+ other than 16/18	
	25	positive	negative	negative	HR+ other than 16/18	33	HR+ other than 16/18	
	26	positive	positive	negative	16 (52)	52	16 (52)	
	27	negative	negative	negative	negative		negative	
	28	negative	negative	negative	16		16	Cobas neg/neg/neg
	29	negative	negative	negative	HR+ other than 16/18	31	HR+ other than 16/18	Cobas neg/neg/neg

Case	Reference test (COBAS)			Study test (E7-HPV)			Confirmatory or Re-test
	12 hr-HPV	hpv 16	hpv 18	Agarose	type	Capillary	
30	positive	negative	negative	negative	neg	negative	Clart neg/neg
31	positive	positive	negative	16	58	16	
32	positive	positive	negative	16 (31)	31	16 (31)	
33	positive	negative	negative	HR+ other than 16/18	31	HR+ other than 16/18	
34	positive	negative	negative	negative	neg	negative	Clart HPV 51
35	negative	negative	negative	negative		negative	
36	negative	negative	positive	18		18	
37	positive	positive	negative	16	35	16	
38	positive	negative	positive	18 (33)	33	18 (33)	
39	negative	negative	negative	negative		negative	
40	negative	positive	negative	16		16	
41	negative	positive	negative	16		16	
42	positive	negative	negative	HR+ other than 16/18	33 52	HR+ other than 16/18	
43	negative	positive	negative	16		16	
44	negative	negative	negative	negative		negative	
45	negative	positive	negative	16		16	
46	negative	positive	negative	16		16	
47	negative	positive	negative	16		16	
48	positive	positive	negative	16 (52)	52	16 (52)	
49	negative	positive	negative	16		16	
50	negative	positive	negative	16		16	
51	positive	positive	negative	16 (52)	52	16 (52)	
52	positive	negative	negative	negative	39	negative	
53	negative	negative	negative	negative		negative	
54	negative	positive	negative	16		16	
55	positive	negative	negative	HR+ other than 16/18	31	HR+ other than 16/18	
56	negative	positive	negative	16		16	
57	positive	negative	negative	HR+ other than 16/18	33	HR+ other than 16/18	
58	positive	positive	negative	16+33	neg	16	Clart HPV 16 / HPV 51
59	positive	negative	negative	HR+ other than 16/18	31	HR+ other than 16/18	
60	negative	positive	negative	16		16	
